# A Case of Double Uterus, with a Fœtus in Each

**Published:** 1885-07

**Authors:** E. W. Lane

**Affiliations:** Scarboro, Ga.


### A CASE OF DOUBLE UTERUS, WITH A FŒTUS IN EACH.

---

READ BEFORE THE MEDICAL ASSOCIATION OF GEORGIA, AT ITS THIRTY-SIXTH ANNUAL SESSION, AT SAVANNAH, APRIL, 1885, BY E. W. LANE, M. D., SCARBORO, GA.

---

On Thursday, the 21st of February, 1884, Mrs. K., age 35, a healthy, robust Georgia lady, *primipara*, was taken with labor pains, and Dr. T. J. Hendley was called in to attend her, and on his arrival made the necessary examination usual in such cases, and found, as he supposed, nothing unusual, only that he could not find the os or feel the fœtus by digital examination. He remained with her almost constantly, and examined her from time to time, until late Saturday night, when he felt a hard substance that he took to be the head. The case progressed so slowly, and was so different from anything that he had ever seen, he asked that I be sent for to assist him, which was very readily agreed to, and a messenger dispatched for me, with a note from Dr. H., asking that I come immediately, and saying, "I think that I have felt the head." I received the note about daylight Sunday morning, and as soon as I could make the necessary preparation I started, but the roads being bad and distant fourteen miles, I arrived there about ten o'clock a. m. On arriving, the Doctor told me that since I had been sent for he thought that he had felt the toes, but was not certain of it, and requested me to examine and see what I thought of it. I accordingly did so, and found, as the Doctor had supposed, a foot presentation, though very high up. After waiting some half hour and finding but little progress in the case, I managed to fix the toes between my index and middle fingers, and at each pain to make traction. In about half an hour's time, we had got the foot to where, with much trouble, we got a ribbon around it, and by making traction on the ribbon at each pain, we got the foot through the vulva. But at this period things seemed to come to a standstill. No traction that we were willing to make would move it a particle. I then introduced my fingers and found that the knee was pressing against some hard substance. At the next pain I pressed it up, at the same time making traction on the ribbon, and the knee slipped by and was brought out. The pains now became stronger, and the balance of the delivery was, as is usual in foot presentations, only more tedious. The child, by being so long in its passage through the parts, proved to be dead. We used all means in our power to resuscitate it, but could only get it to gasp twice. It was born about one o'clock p. m. The child, a male, was larger than an average. We were not long in discovering that there was another fœtus in utero. We ligated the cord and divided below and left the patient to rest, but as the pains were very feeble and the patient much exhausted, we gave her a stimulant, shortly after which they began to improve a little. I then made a digital examination by following up the cord. My finger passed into the uterus, but could find no presenting fœtus, but being very confident that there surely was another, I again explored the organ as far as my finger could reach, but by feeling about I found a hard substance to my right, probably the same that Dr. Hendley had felt before having had me sent for, and by further examination found the os dilated about the size of a twenty-five cent piece, opening laterally into the vagina. We waited all the balance of Sunday and Sunday night, and until a little after sunrise Monday morning. The bag of waters by that time was pressing open the vulva. We then ruptured it, hoping that the pains would become more expulsive, but it seemed to do no good in that respect. We waited until about eight o'clock a. m., and finding that the head had become sufficiently low down, and that the fœtus was dead, we punctured its head, and I hooked my fingers in the head and delivered a female child of medium size. It had been dead probably for the last twenty hours or more. There was but little hemorrhage. Both placentas were retained. I introduced my hand and followed the cord of last delivered fœtus to the placenta and delivered it. The uterus contracted as I withdrew it. I then followed the other cord, which led my hand into another uterine cavity, and I found the placenta adherent. I detached it and removed it, but the uterus did not contract, and the hemorrhage was considerable. I again introduced my hand, and with a cloth in it about the size of a ladies' pocket handkerchief, saturated in apple vinegar, and thoroughly explored the organ, and near the fundus I found a semi-solid substance, nearly or quite as large as either of the placentæ. I detached and removed it, and uterus No. 2 contracted, forcing out my hand with what I took to be a male uterus. No. 2 was then well contracted, and both could be felt through the walls of the abdomen, distinctly separated and lying side by side. Both placentæs were attached to the right side of its uterus.

The lady lay in a comatose condition until two o'clock p. m. Monday, when she rallied ; and from then until her final getting up, which was in usual time in ordinay cases, there was nothing more in her case worthy of note.

