# Correction

**Published:** 1885-08

**Authors:** 


CORRECTION.—In the July issue of THE JOURNAL, page 277, an absurd typographical error occurs. The error occurs in line 28, where No. 2 should read *No. 1*. Line 29, male should read *mole*. The phrase corrected should read: "I detached and removed it, and uterus No. 1 contracted, forcing out my hand with what I took to be a mole. Uterus No. 2 was then well contracted, and both could be felt through the walls of the abdomen, distinctly separated and lying side by side."

